# Performance of Asphalt Materials Based on Molecular Dynamics Simulation: A Review

**DOI:** 10.3390/polym17152051

**Published:** 2025-07-27

**Authors:** Chengwei Xing, Zhihang Xiong, Tong Lu, Haozongyang Li, Weichao Zhou, Chen Li

**Affiliations:** 1 Key Laboratory for Special Area Highway Engineering of Ministry of Education, Chang’an University, South 2nd Ring Road Middle Section, Xi’an 710064, China; xingcw@chd.edu.cn (C.X.); xiongzh@chd.edu.cn (Z.X.); lihzy@chd.edu.cn (H.L.); zhouwc@chd.edu.cn (W.Z.); yaozichen666@163.com (C.L.); 2 School of Highway, Chang’an University, South 2nd Ring Road Middle Section, Xi’an 710064, China

**Keywords:** molecular dynamics simulation, asphalt binder, molecular modeling, self-healing, aging, interfacial adhesion, asphalt modifiers

## Abstract

With the rising performance demands in road engineering, traditional experiments often fail to reveal the microscopic mechanisms behind asphalt behavior. Molecular dynamics (MD) simulation has emerged as a valuable complement, enabling molecular-level insights into asphalt’s composition, structure, and aging mechanisms. This review summarizes the recent advances in applying MD to asphalt research. It first outlines molecular model construction approaches, including average models, three- and four-component systems, and modified models incorporating SBS, SBR, PU, PE, and asphalt–aggregate interfaces. It then analyzes how MD reveals the key performance aspects—such as high-temperature stability, low-temperature flexibility, self-healing behavior, aging processes, and interfacial adhesion—by capturing the molecular interactions. While MD offers significant advantages, challenges remain: idealized modeling, high computational demands, limited chemical reaction simulation, and difficulties in multi-scale coupling. This paper aims to provide theoretical insights and methodological support for future studies on asphalt performance and highlights MD simulation as a promising tool in pavement material science.

## 1. Introduction

In recent years, with the development of high-grade road engineering and the continuous increase in traffic load, the demand for performance research on asphalt materials has become increasingly urgent [1]. Therefore, a deeper understanding of the relationship between the physicochemical properties of asphalt and its molecular composition is required [2]. However, asphalt is a black-brown mixture primarily composed of hydrocarbons with varying molecular weights and their non-metallic derivatives, including heteroatoms such as oxygen, sulfur, and nitrogen [3,4,5,6]. This complex chemical composition and molecular structure make it difficult for traditional experimental methods to accurately characterize the molecular makeup of asphalt. With advancements in computer science and technology, molecular simulation has provided new possibilities for analyzing the performance of asphalt materials at the nanoscale [7,8,9,10]. Common molecular simulation approaches include quantum mechanics (QM), molecular mechanics (MM), molecular dynamics (MD), and Monte Carlo (MC) methods [11]. Among these, MD simulation has become the most widely used tool for simulating the dynamic behavior of asphalt molecules, as it can accommodate multi-component systems through coarse-grained models [12].

**Figure 1:**
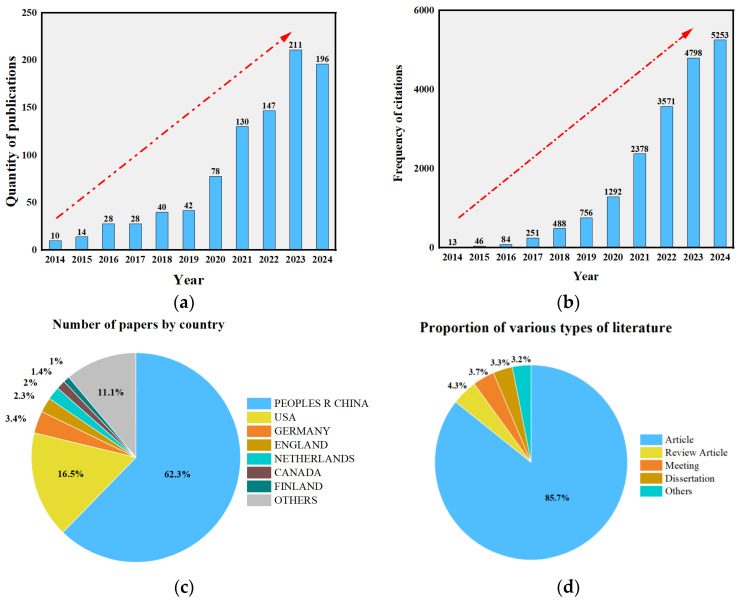
Analysis of related papers: (**a**) chart of the number of years of the publication literature (**b**) frequency of citations (**c**) number of papers by country (**d**) number of document types.

In recent years, the molecular simulation software *Materials Studio* version 2020 (MS) has gained increasing attention. A search using the keywords “molecular dynamics” and “asphalt” in the Web of Science Core Collection retrieved a total of 938 relevant English-language publications. Based on the analysis of the search results and the generation of a citation report, *HistCite Pro 2.1* was further employed to statistically evaluate these publications and their citation patterns. As shown in Figure 1a, the number of publications has exhibited a steady upward trend since 2014, indicating a growing interest in and adoption of MD simulation methods among researchers. Figure 1b demonstrates that citation frequencies of relevant papers began to increase significantly from 2015 onward, with a particularly sharp rise observed between 2018 and 2023. These data clearly reflect the progressive maturation of asphalt research based on MD simulation over the past five years.

Furthermore, citation analysis using the Graph Maker function of *HistCite Pro 2.1* revealed that the publication with the highest Local Citation Score (LCS) was published in 2014. Overall, the majority of highly cited papers (with high LCS values) were concentrated between 2014 and 2022, indicating that these studies have laid a solid theoretical foundation and provided directional guidance for subsequent research. Figure 1c presents the country-wise distribution of the relevant publications. The results show that China and the United States lead significantly in the number of publications in this field, accounting for 62.3% and 16.5%, respectively. This trend highlights the strong research capacity and international influence of the scholars from these two countries in asphalt materials research. In addition, Germany and the United Kingdom, along with other Asian regions (such as Japan and South Korea), have also made important contributions. A further analysis of the document types, as shown in Figure 1d, indicates that research articles constitute the overwhelming majority. This suggests that the application of molecular dynamics simulation in asphalt materials remains in an active phase of exploration and is expected to demonstrate even greater research potential in the future.

As a key technique for analyzing the microscopic behavior of asphalt materials, MD simulation has become an essential tool for studying asphalt performance at the molecular level and plays a critical role in understanding its macroscopic properties as well [13]. By constructing molecular models, MD simulations enable in-depth investigation of the microstructure and performance characteristics of asphalt materials, as well as elucidation of the mechanisms and effects of various modifiers on asphalt modification [7]. Currently, most studies utilize MD simulation in conjunction with microstructure–property correlation analyses to explore the behavior of asphalt binders and their modifiers. However, a comprehensive and systematic summary of the mechanisms revealed by MD simulations in this context remains limited. This lack of integration restricts the broader application of MD in deeper investigations, highlighting the academic value of developing integrative reviews and methodological overviews in this field.

This review systematically summarizes the latest advances in applying MD simulations to the study of asphalt material performance and its underlying microscopic mechanisms, aiming to provide a structured reference and theoretical guidance for future research. It begins with an introduction to the basic principles of MD, detailing its unique advantages in modeling intermolecular interactions and predicting material properties. The core functions and workflows of the relevant software, such as *Materials Studio 2020*, are also described. Subsequently, the review traces the development of asphalt molecular models, modifier molecular models, and asphalt mixture models, from early simplified versions to more recent complex and precise structures. It systematically outlines improvements and optimizations in model construction methods, particularly the practical techniques and implementation steps using *Materials Studio*, showcasing the continuous advancement of MD simulations in asphalt research. Furthermore, the review integrates studies related to the performance of base asphalt, modified asphalt, and asphalt mixtures from a molecular perspective. By exploring the intrinsic relationships among molecular interactions, modification mechanisms, and macroscopic performance, the review highlights the critical role of MD in performance optimization and mechanism characterization. Finally, the paper discusses the main challenges facing MD simulation in asphalt research, including the limitations in model accuracy, high computational complexity, and difficulties in multi-scale coupling. Based on the recent research trends, it also proposes future directions for the field, such as simulations of complex multi-component systems, deeper exploration of the modifier interaction mechanisms, and integration with experimental data. These perspectives aim to further advance the application of MD techniques in asphalt materials research and provide new insights for the continued development of this field.

## 2. Molecular Models of Asphalt, Modifiers, and Asphalt–Aggregate Interfaces

### 2.1. Generic Model Method

**Figure 2:**
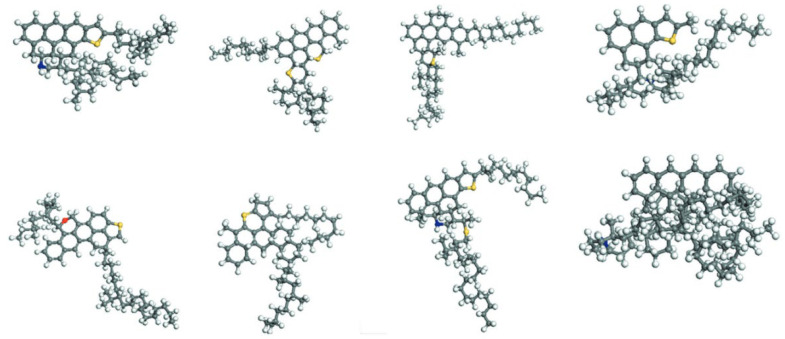
Eight average molecular models for bitumen binders [2,15].

The generic model molecular model of asphalt was developed to simplify the complex chemical nature of asphalt by representing it with a set of typical molecular structures. Essentially, this approach captures the overall average chemical characteristics of asphalt through a limited number of representative molecular configurations. The development of average asphalt models has undergone a lengthy evolution. Among early studies, Jennings et al. [14], based on nuclear magnetic resonance (NMR) spectroscopy, identified the key structural features such as aromaticity, aliphatic chain length, and the presence of nitrogen- and sulfur-containing functional groups. Based on these findings, they proposed eight standardized molecular models of asphalt samples, which laid the foundation for the subsequent research on the asphalt molecular structure. These models were developed within the Strategic Highway Research Program (SHRP). They include AAA-1, AAB-1, AAC-1, AAD-1, AAF-1, AAG-1, AAK-1, and AAM-1. Each model represents an average molecular structure of asphalt. Researchers used these models to study the properties of asphalt in detail. [2,15]. Figure 2 shows these molecular structures. These models encompass asphalts from different sources and with varied performance characteristics, providing a foundational basis for the molecular simulation research on asphalt. With respect to specific molecular characteristics, studies have found that polar aromatics typically exist as black solids at room temperature [16] and possess lower molar masses than asphaltenes, although their molecular compositions are very similar [17]. Building upon these insights, Murgich et al. [18] proposed an average molecular structure model for polar aromatics, offering a valuable reference for investigating the microscopic properties and intermolecular interactions of asphalt. The development of average molecular models has not only established the theoretical basis for constructing asphalt microstructural models but has also introduced a novel approach to studying the relationship between the molecular structure and material performance. These models reveal the distribution characteristics of the key components within asphalt and underscore the necessity of incorporating substitutable atoms, thereby offering a clear direction for the further optimization of asphalt molecular models.

### 2.2. Molecular Assembly Method

#### 2.2.1. Three-Component Model

**Figure 3:**
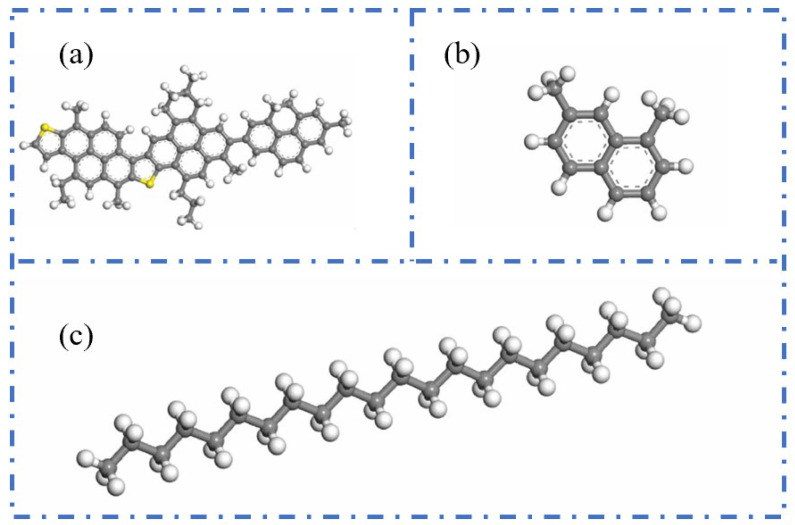
Molecular structures of three components of asphalt binder. (**a**) Asphaltenes; (**b**) resins; (**c**) n-C22 (n-docosane) [22].

The three-component approach classifies asphalt into three main constituents—oils, resins, and asphaltenes—which serves as the foundation for constructing asphalt molecular models. Groenzin and Mullins [19] were the first to propose a molecular structure for asphaltenes. Building upon this, Zhang and Greenfield [19,20] developed representative molecular models for resins and oils, namely the 1,7-dimethylnaphthalene molecule and the n-C22 (n-docosane) molecule. Based on these studies, Ding et al. [21] constructed a three-component molecular model of asphalt using the aforementioned molecular structures for asphaltenes, resins, and oils. This model provides an intuitive framework for investigating the microscopic behavior of asphalt. The specific configuration of the three-component model is illustrated in Figure 3.

#### 2.2.2. Four-Component Model

**Figure 4:**
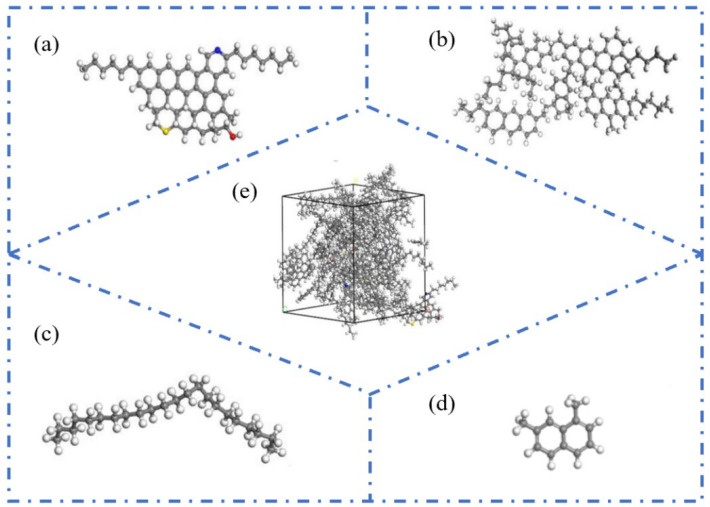
Four-component molecular model of asphalt. (**a**) Pyrrole; (**b**) molecules derived from maltene condensation reactions; (**c**) 1,7-dimethylnaphthalene; (**d**) n-docosane (n-C22); (**e**) virgin asphalt [6].

With the deepening understanding of the asphalt molecular composition, researchers have identified certain limitations in the three-component approach when it comes to comprehensively representing asphalt’s molecular structure. Specifically, the study by Rostler et al. [23] indicated that a single average molecular model is insufficient to fully characterize the diverse properties of asphalt. In contrast, the four-component model (aromatics, saturates, resins, and asphaltenes) provides a more refined and detailed depiction of asphalt’s molecular architecture. Based on this theory, Dong [24] and Qi [25] proposed molecular models for asphaltenes and resins. Further, Li et al. [6] successfully constructed a four-component molecular model of base asphalt (as shown in Figure 4), providing critical support for the further exploration of the relationship between asphalt’s molecular structure and its macroscopic performance. The four representative average molecular structures correspond to the key components of asphalt. Specifically, resins, aromatics, saturates, and asphaltene are represented by the molecules derived from maltene condensation reactions [26], 1,7-dimethylnaphthalene, n-docosane (n-C22) [27], and pyrrole.

**Figure 5:**
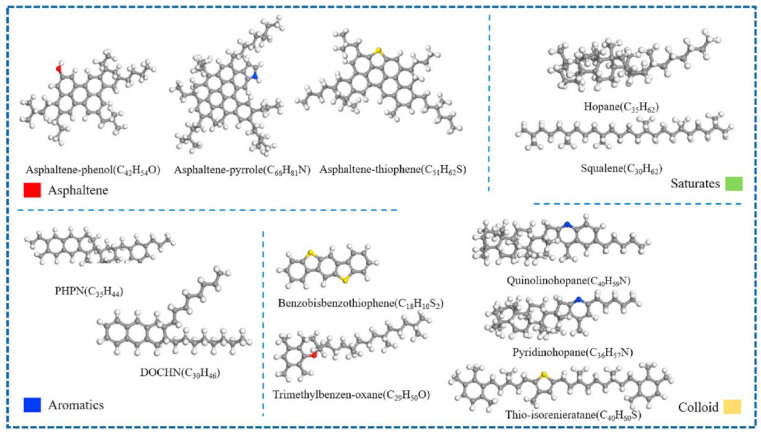
Four-component 12-molecule model of asphalt [31].

**Figure 6:**
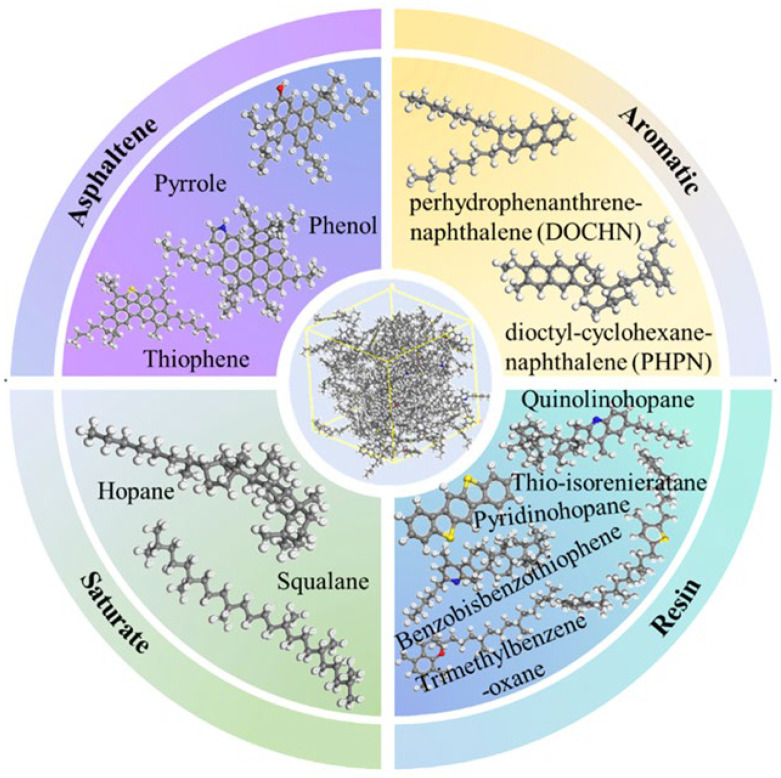
AAA-1 molecular model of asphalt [32].

**Figure 7:**
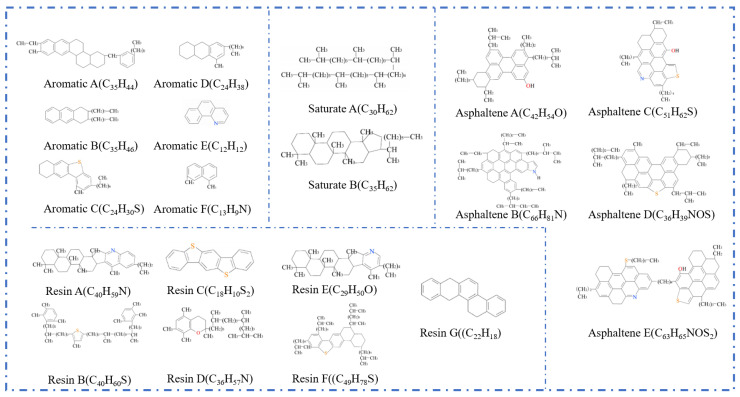
Twenty-molecule model of asphalt [30].

With the continuous advancement of asphalt molecular modeling, Li and Greenfield [28] proposed three types of four-component 12-molecule models. These models are named AAA-1, AAK-1, and AAM-1. The models are based on the mass percentages of carbon, hydrogen, oxygen, nitrogen, and sulfur in base asphalt. They also consider the atomic hydrogen-to-carbon (H/C) ratio. The molecular composition of each model is illustrated in Figure 5, with the detailed structure of AAA-1 shown in Figure 6. These models offer a more accurate representation of the physical and mechanical properties of asphalt. However, due to the bond conflicts among molecules, the pentane effect can occur, causing asphaltene molecules to exhibit high internal energy, which negatively impacts the model stability. To address this issue, Li et al. [29] optimized the models by adjusting the positions of alkyl chains, significantly reducing the internal energy and thereby improving both model stability and practical applicability. The optimized models better reflect the actual properties of asphalt and are considered among the most advanced and widely studied models in current asphalt research. Nonetheless, limitations remain. The model has primarily been validated and applied to the AAA-1 asphalt type. A core challenge is that molecular compositions vary significantly among asphalts from different sources, making it difficult to establish a direct correlation between simulation results and experimental data. To overcome this bottleneck, researchers have continued to refine the asphalt molecular modeling methods. Based on extensive experimental data and the molecular structural characteristics of asphalt, Xu et al. [30] innovatively constructed three representative molecular models. Key parameters such as density, maximum adhesion force, and modulus were obtained through simulations. Comparative analysis with experimental data verified the reliability of the models. However, some limitations remain. The validation system still lacks important dynamic parameters, such as viscosity and diffusion coefficients. In addition, the model contains 20 molecular structures, which results in high computational demands, as shown in Figure 7.

In a separate study, Yao et al. [8] improved upon the four-component model by introducing 12 distinct molecules (such as quinoline and thia-pyrene), achieving a chemical composition more consistent with real asphalt. This refinement reduced the density error to 0.06 g/cm^3^ and significantly enhanced the accuracy of predictions for the solubility parameters and viscosity. Similarly, Liu et al. [33] employed a 14-molecule model to investigate the formation mechanisms of molecular nano-micelles and intrinsic π–π interactions in SBS-modified asphalt. While many such examples exist, the 12-molecule four-component model remains the most widely adopted in the field, offering a balance between simulation accuracy and computational efficiency.

In asphalt molecular dynamics (MD) simulations, selecting an appropriate molecular model is essential for uncovering microscopic mechanisms. The predominant approaches include the three-component model and the twelve-molecule four-component model, each offering distinct strengths and limitations. A systematic comparison of their applicability is thus warranted.

The three-component model—comprising asphaltenes, resins, and saturates—is valued for its simplicity and efficiency. It is well-suited for preliminary studies of macroscopic properties such as diffusion and thermal stability, particularly in fundamental or comparative research. However, by omitting aromatic fractions and polarity differences, it struggles to capture complex molecular interactions accurately. The four-component model builds upon this by incorporating greater diversity into aromatics and resins, forming a system of asphaltenes, resins, aromatics, and saturates. This model more faithfully represents asphalt’s chemical composition and yields improved predictions of density, solubility, self-healing, and aging behavior. Its complexity, however, entails higher computational costs.

Overall, the three-component model is ideal for efficient, exploratory simulations, while the four-component model enables more accurate analyses of structure–property relationships and is preferred for detailed modeling. The model selection should align with the research objectives, and future developments may benefit from multi-scale or modular strategies integrated with experimental validation. To provide a more concise comparison of the comprehensiveness of the three-component and four-component models, Table 1 presents a summary of their key features.

### 2.3. Common Modifier Models

#### 2.3.1. SBS Modifier Models

**Figure 8:**
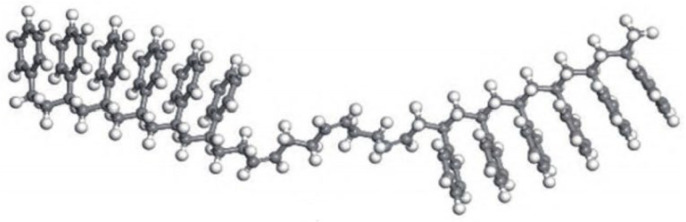
Molecular model of the SBS modifier [38].

**Figure 9:**
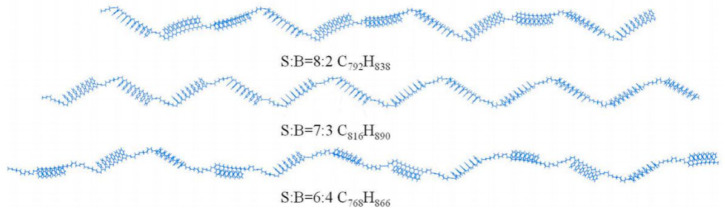
Molecular models of SBS modifiers with different block ratios [40].

Commonly used polymer modifiers for asphalt include SBS, polyethylene, polypropylene, and ethylene-vinyl acetate copolymer (EVA), among which SBS-modified asphalt is the most widely applied [34]. Styrene–butadiene–styrene block copolymer belongs to thermoplastic elastomers and is the most commonly used high-viscosity modifying component in engineering applications [35,36]. During the mixing process, SBS and asphalt gradually form a microscopic cross-linked structure, significantly enhancing the pavement performance of the modified asphalt mixture [37]. The molecular weight of SBS is 10^5^, and representative molecular segments are generally selected for model construction [38]. According to related studies [9,39], Fan et al. [38] constructed an SBS molecular model using MS 2020 software, mainly composed of six styrene units, three butadiene units, and six styrene units polymerized sequentially, as shown in Figure 8. SBS is a long-chain thermoplastic elastomer polymer modifier. Meanwhile, the block ratio is a key parameter during the modification process. Li, G. et al. [40] constructed three typical linear SBS molecules with different block ratios, as illustrated in Figure 9, where each SBS molecule contains six different concentrations ranging from 2% to 12% with 2% intervals. Of course, SBS can be categorized into linear SBS (L-SBS) and star-shaped SBS (S-SBS). Mouillet et al. [41] observed via microscopy that L-SBS appears as dispersed spherical particles in modified asphalt, whereas S-SBS forms irregular shapes.

#### 2.3.2. SBR Modifier Model

**Figure 10:**
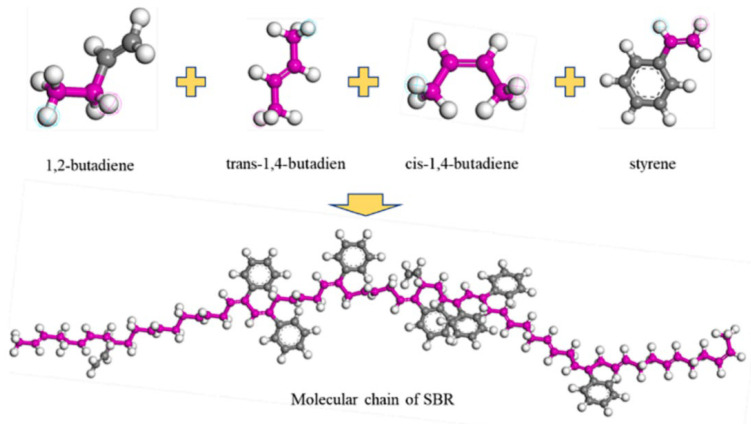
SBR molecular model [46].

**Figure 11:**
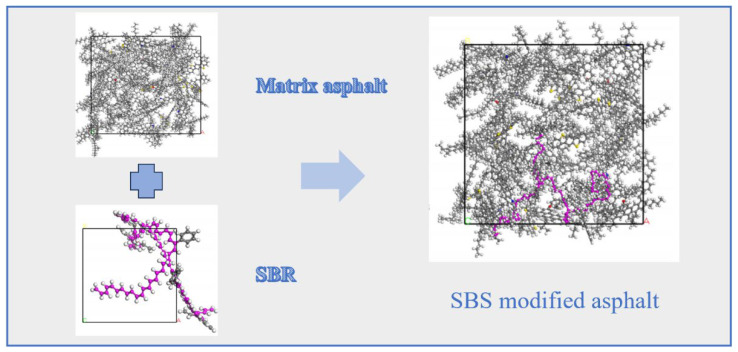
Crystal cell model of asphalt and the SBR modifier [47].

A large number of used tires are produced worldwide each year. The traditional disposal methods for these waste tires include incineration, landfilling, and storage, all of which undoubtedly pose environmental hazards [42,43,44]. According to the study by Liao et al. [45], the main component of tires is SBR, a random copolymer derived from the monomers, styrene and butadiene. Based on previous studies, Xie T. et al. [46] constructed an SBR polymer model, as shown in Figure 10. In their investigation into the fundamental mechanism underlying the compatibility between the SBR modifier and base asphalt, Jiao X. L. et al. [47] established crystalline unit models of asphalt and the modifier, as illustrated in Figure 11.

#### 2.3.3. PU Modifier Model

**Figure 12:**
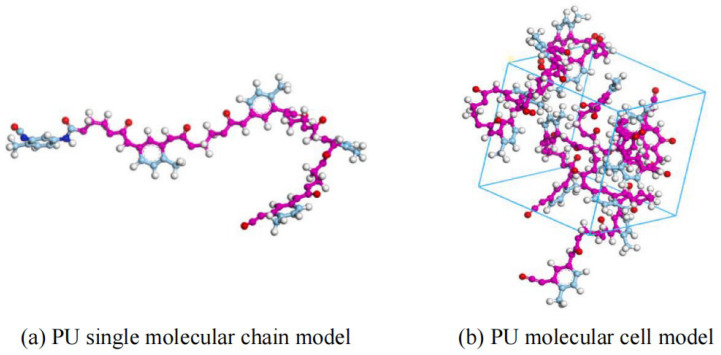
PU molecular model [49].

**Figure 13:**
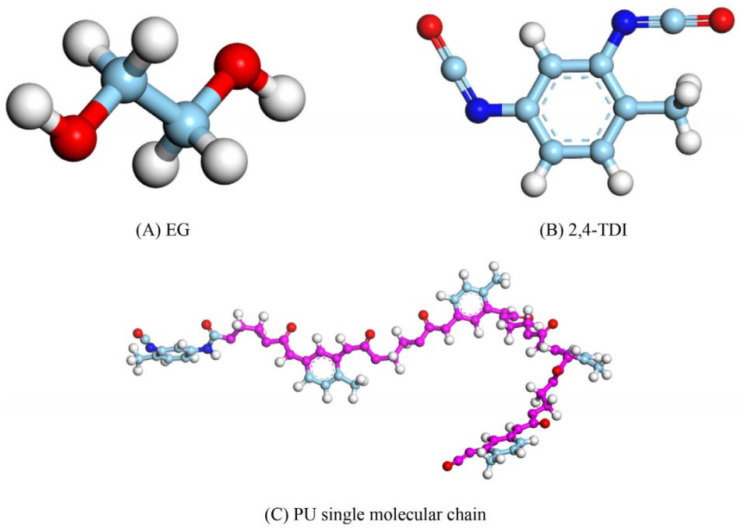
Molecular model of PU [50].

**Figure 14:**
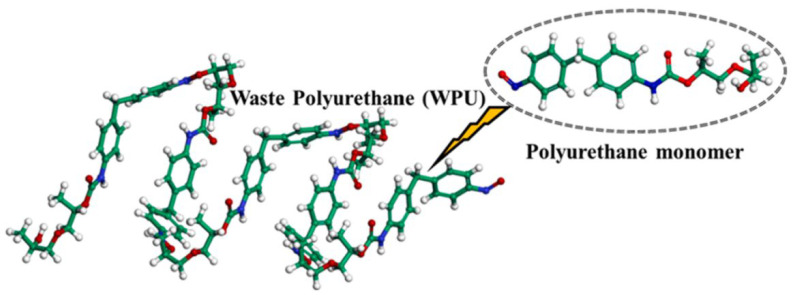
Waste polyurethane molecular chain [51].

PU is a novel polymer modifier that contains the –NHCOO– structure in its main chain. Due to its good compatibility with asphalt, PU is commonly used as an asphalt modifier [48]. Considering the high molecular weight of PU and the need for accurate and reasonable simulation, Lu P. et al. [49] selected a single-chain PU model with a degree of polymerization of 4, in which the urethane segments are repeated up to 10 times. The single-chain PU model (a) and the complete PU molecular model (b) are shown in Figure 12. Lu P. et al. [50] further investigated the mechanical properties of PU, using polyethylene glycol (PEG) as the soft segment and 2,4-toluene diisocyanate (2,4-TDI) as the hard segment. The polymer model was constructed based on the monomers, ethylene glycol and 2,4-TDI, as illustrated in Figure 13. On the basis of PU modifiers, Zhang T. et al. [51] explored the effects of waste polyurethane (WPU) on the rheological properties and compatibility of SBS-modified asphalt. The molecular models of WPU and WPU/SBS-modified asphalt were established, as shown in Figure 14.

#### 2.3.4. PE Modifier Model

**Figure 15:**
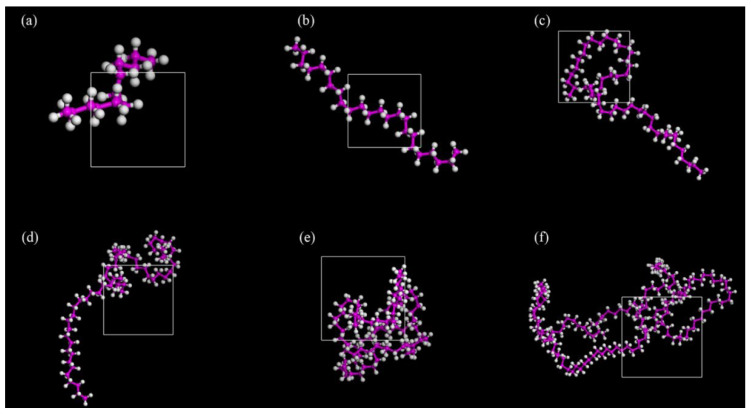
PE molecular model with different degrees of polymerization (**a**) 6 (**b**) 12 (**c**) 24 (**d**) 30 (**e**) 42 (**f**) 78 [53].

**Figure 16:**
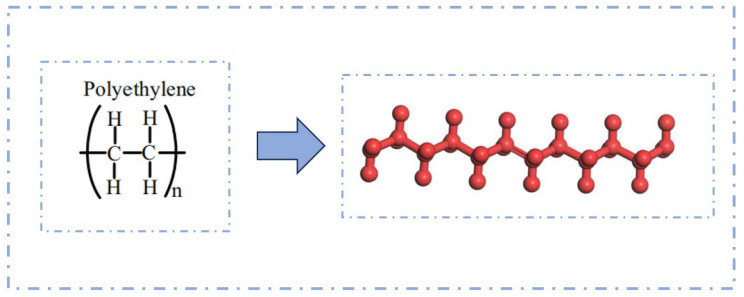
PE modifier model [54].

**Figure 17:**
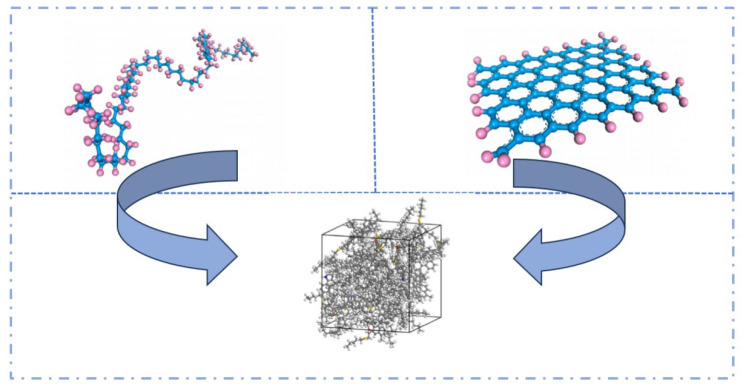
Graphene/RPE–modified asphalt models [55].

PE is a cost-effective asphalt modifier that offers an economical and efficient solution for improving pavement performance [52]. Chen Y. et al. [53] investigated the interaction mechanism of plastic-modified asphalt at the molecular level and established molecular models of PE modifiers with varying numbers of molecules to determine the optimal degree of polymerization (see Figure 15). The results indicated that the optimal degree of polymerization for PE molecules is 12. To further explore the performance of PE-modified asphalt, Li E. D. et al. [54] constructed a polyethylene molecular model based on its molecular formula. They then evaluated the compatibility between asphalt and PE by calculating the solubility parameters and interaction energies, with the established model shown in Figure 16. On the basis of PE-modified asphalt, Hu K. et al. [55] investigated the reinforcing mechanism of graphene on recycled polyethylene (RPE)-modified asphalt, and the corresponding model is illustrated in Figure 17.

### 2.4. Molecular Dynamics Simulation of Phase Separation and Compatibility in Polymer-Modified Asphalt

Asphalt is a multi-component material with significant polarity differences among its constituents. When polymer modifiers such as SBS, SBR, PE, or PU are added, phase compatibility and separation become the key factors affecting the modification efficiency and service stability. Traditional experimental methods cannot directly observe molecular-level interfacial behavior, whereas MD simulations offer a powerful tool to explore the interactions between polymers and the asphalt matrix.

Firstly, MD simulations can quantitatively evaluate the compatibility between modifiers and various asphalt components by calculating the solubility parameters and interaction energies. Jiao X. L. et al. [47] calculated the interaction energy between SBR and the asphalt matrix using MD simulations, finding that the interaction energy reaches its minimum value at 160 °C. This indicates the optimal compatibility of the SBR–asphalt system at this temperature, with van der Waals forces being the dominant interaction. Su M. et al. [56] used MS software to calculate the solubility parameters and molecular structural parameters of SBS-modified asphalt, and employed microscopy techniques to observe its morphological characteristics. They found that the compatibility results between asphalt and SBS from experimental detection closely matched the simulation outcomes. Moreover, Li N. et al. [57] evaluated the effect of the compatibilizer content on the system’s compatibility by quantifying the cohesive energy density (CED) and solubility parameters. Radial distribution function (RDF) and mean square displacement (MSD) analyses were conducted to reveal the molecular diffusion dynamics, and the accuracy of the model was validated using atomic force microscopy (AFM). In addition, He L. et al. [58] developed a coarse-grained molecular dynamics (CGMD) model and corresponding force field for asphalt materials, overcoming the scale limitations of previous studies. This approach enabled the simulation of the evolution of polymer phase structures and provided deeper insights into the microscopic mechanisms of asphalt self-healing.

Overall, MD simulations often assume an ideal, uniformly distributed initial state, which cannot fully reflect the complex phase behavior caused by shear, thermal stress, and oxidative aging in real conditions. Thus, more detailed models combined with experimental data (e.g., AFM and TEM) are needed to better understand the compatibility and phase evolution of polymers in asphalt.

### 2.5. Molecular Models of Asphalt–Aggregate Interaction

**Figure 18:**
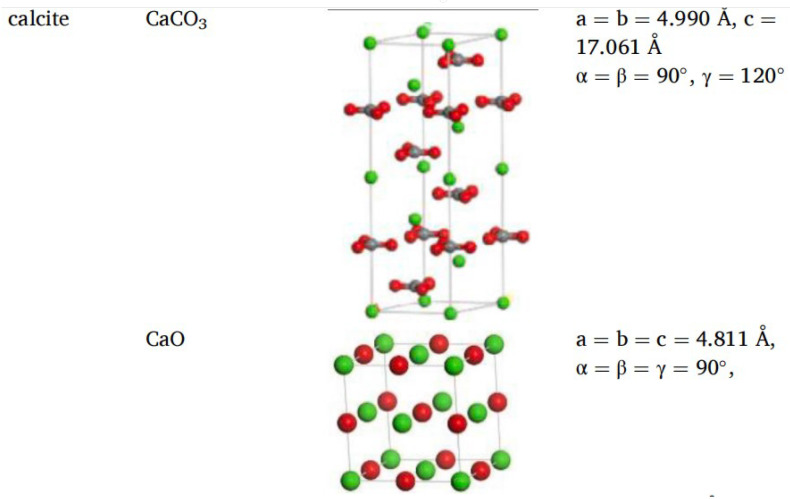
Unit cells of silicon dioxide and calcium oxide [61,62].

**Figure 19:**
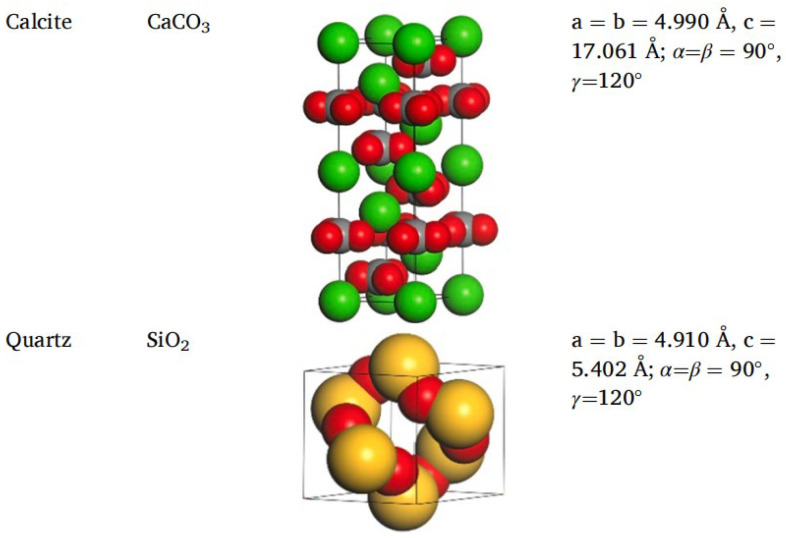
Unit cell structure and lattice parameters of calcite [63].

**Figure 20:**
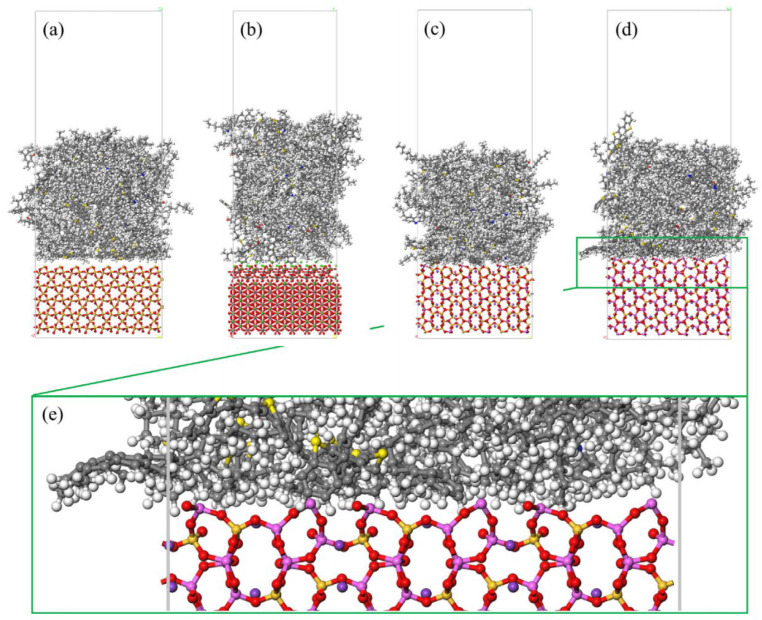
Mineral–bitumen interface systems after MD simulations: (**a**) quartz–asphalt model; (**b**) calcite–asphalt model; (**c**) albite–asphalt model; (**d**) microcline–asphalt model; and (**e**) locally enlarged interface structure for microcline–asphalt model [64].

Different rocks exhibit distinct structures and characteristics. Horgnies, Darque-Ceretti, Fezai, and Felder [59] studied the mineral composition of rocks using X-ray fluorescence spectroscopy and investigated the effects of these mineral components on the tensile strength of aggregates. Their results indicated that SiO_2_, Al_2_O_3_, CaO, MgO, and Fe_2_O_3_ are the key mineral constituents influencing aggregate performance. In molecular simulations, aggregate models are typically represented by oxide unit cells, with common aggregate types including calcium oxide and silicon dioxide. Calcium oxide, the most common type of aggregate used in asphalt mixtures, is an alkaline aggregate with calcite as its crystalline form; therefore, calcite is often selected as the representative mineral for constructing calcium oxide aggregate models [60],while SiO_2_ represents acidic aggregates. By establishing unit cells for two types of silicon dioxide and calcium oxide [61,62], the adsorption process between asphalt and aggregate was simulated, revealing that the polarity effects between asphalt and aggregate are the primary cause of adhesion, as shown in Figure 18. Chen P. et al. [63] derived the crystal structure of calcite from the Cambridge Structural Database (CSD), with its unit cell structure and lattice parameters illustrated in Figure 19. To further investigate the influence of mineral types and water on the adhesion performance of the asphalt–mineral interface system, Gao et al. [64] selected four representative minerals (quartz, calcite, albite, and orthoclase) and constructed mineral–asphalt and mineral–water–asphalt interface systems, as depicted in Figure 20.

## 3. Molecular Dynamics Simulation for the Study of Asphalt Material Properties

### 3.1. High-Temperature Performance of Asphalt

The poor thermal stability of asphalt has become a critical factor limiting the long-term durability of asphalt pavements worldwide. In response, many high-grade highways have adopted modified asphalt binders to enhance the thermal stability and overall performance of pavement materials [65]. Li G. et al. [66] revealed that the high-temperature performance of hard asphalt is primarily governed by the resin content and the number of saturated carbons, microscopically relying on the stability of short-range disordered structures. Process optimization should focus on component compatibility to avoid aggregation. Hou et al. [67] analyzed the stress characteristics of asphalt components using molecular dynamics methods and found that high-stress regions exist at the interface between the saturate and cycloalkane-aromatic phases. This high stress sharply increases from the center of the cycloalkane-aromatic phase toward a specific direction at the interface (e.g., left or right). The results indicate that compared to the cycloalkane-aromatic phase, the saturate phase plays a more critical role in the micro-mechanical behavior of asphalt. Hu Q. et al. [68] predicted the viscosity variations of reclaimed asphalt binder at elevated temperatures using molecular dynamics (MD) simulations. The simulated viscosity values showed a deviation of less than 11.2% from the experimental results, demonstrating the high reliability and feasibility of the model in predicting high-temperature viscosity. Song M. et al. [69] utilized molecular dynamics (MD) simulations to investigate the rheological behavior and modification mechanism of SiC-modified asphalt. The findings revealed that SiC ceramic microparticles, characterized by their high specific surface area and porous structure, effectively adsorbed the lighter components of asphalt, thereby significantly enhancing its viscosity and high-temperature stability. Furthermore, Sultana et al. [70] measured the dynamic shear modulus and tensile strength of asphalt binders using dynamic shear rheometry (DSR) and tensile compression tests, respectively, and analyzed the microscopic mechanisms through molecular dynamics simulations. The simulation results demonstrated that asphalt with higher resin and asphaltene contents exhibits greater stiffness and tensile strength.

**Figure 21:**
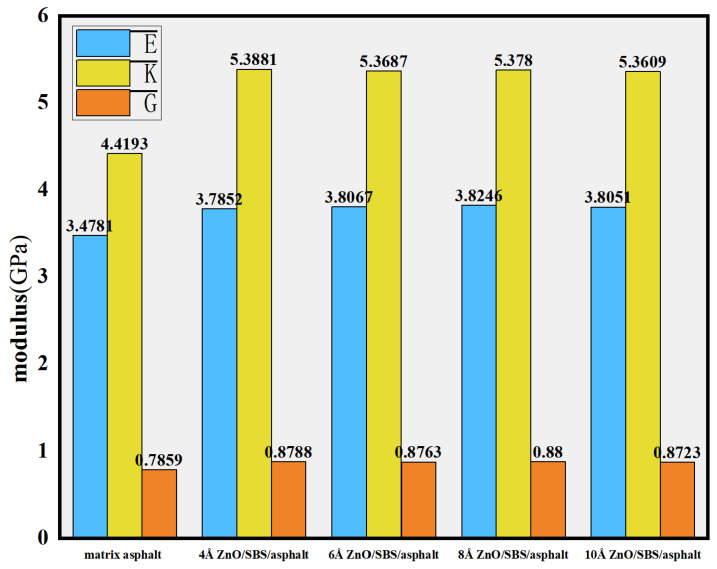
Physical modulus of nano-ZnO/SBS-modified asphalt: elastic modulus (E), bulk modulus (K), and the shear modulus (G) [9].

Previous studies have indicated that crumb rubber and SBS modifiers exhibit significant effects in enhancing the high-temperature performance of base asphalt and are thus being widely used to improve pavement performance [71]. Su M. et al. [9] employed molecular dynamics simulations to investigate the effects of SBS modifiers and ZnO/SBS composite modifiers with different nanoparticle sizes on the high-temperature performance of base asphalt. The mechanical properties of zinc oxide, SBS, and asphalt were analyzed within the temperature range of 383.15 K to 453.15 K, focusing on the influence of ZnO nanoparticles with diameters of 4 Å, 6 Å, 8 Å, and 10 Å. The results demonstrated that ZnO nanoparticles with a diameter of 8 Å yielded the best overall performance improvement (see Figure 21). The enhancement of the bulk modulus was most significant, indicating that the modified asphalt possesses better stability under high temperature and shear conditions.

### 3.2. Low-Temperature Performance of Asphalt

**Figure 22:**
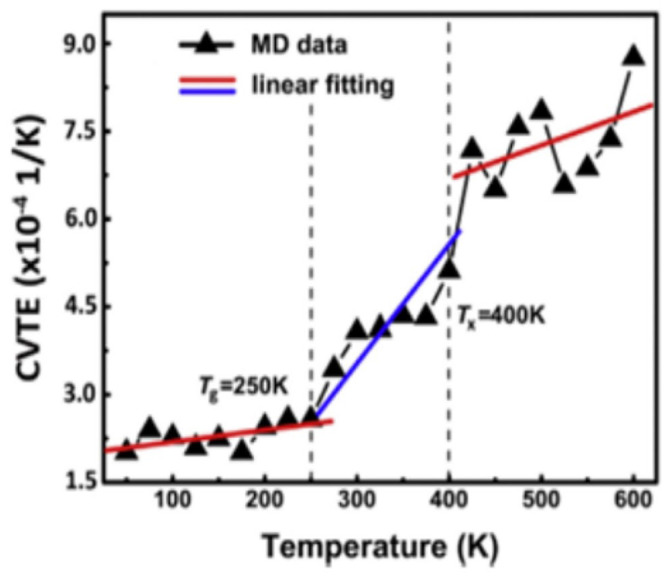
Specific volume versus temperature curve of the AAA-1 asphalt model [75].

**Figure 23:**
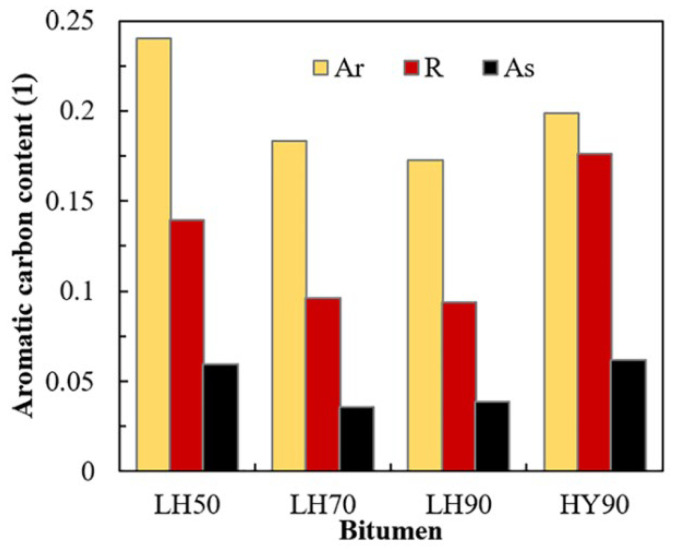
Aromatic carbon content of aromatics (Ars), resins (Rs), and asphaltenes (Ass) [76].

In low-temperature environments, asphalt pavements are susceptible to cracking and structural deformation caused by thermal contraction, which compromises their overall structural integrity and significantly reduces their service performance and durability [72]. To improve the performance of asphalt pavements under low-temperature conditions, high-grade base asphalts are commonly employed. In China, 70# asphalt is widely used in southern regions, while 90# asphalt is more frequently adopted in northern areas. Asphalt mixtures prepared with high-grade binders exhibit a lower low-temperature stiffness modulus, thereby reducing the risk of cracking under cold weather conditions [73]. With the advancement of molecular simulation techniques, the understanding of the molecular mechanisms governing asphalt behavior at low temperatures has deepened. The low-temperature performance of asphalt depends on its chemical composition and molecular structure. Cao et al. [74] used the glass transition temperature *(Tg)* to evaluate the low-temperature performance of SBS-modified asphalt and validated the corresponding experimental methods, including dynamic shear rheometer (DSR) tests, at the molecular level. Their results showed a good correlation between the physical measurements of *Tg* and macroscopic test results, indicating that *Tg* can be used to assess the low-temperature performance of SBS-modified asphalt. The AAA-1 base asphalt model exhibited a glass transition temperature around 250 K when the temperature was lowered from 400 K to 150 K, as shown in Figure 22 [75]. Using differential scanning calorimetry (DSC), Liu S. et al. [76] measured the glass transition temperatures of four asphalt samples with different penetration grades (penetration grades from 50 to 90), with the results illustrated in Figure 23. They found that base asphalts with higher average diffusion coefficients demonstrate better low-temperature performance, attributed to their increased molecular mobility. By combining molecular dynamics simulations with asphalt compositional analysis, Liu S. et al. [76] further discovered that the increase in aromatic carbon in resins and aromatic fractions promotes the dispersion of asphaltene and resin aggregates, thereby improving the low-temperature performance. Specifically, a higher aromatic carbon content reduces the aggregation of asphaltenes and resins, indicating that asphalts with a higher aromatic carbon content exhibit superior low-temperature properties. The molecular simulation results are consistent with the experimental glass transition temperature findings, demonstrating that molecular dynamics simulations can effectively predict the low-temperature performance of asphalt. Qu X. et al. [77] investigated the effects of different paraffin contents on the low-temperature stability, self-healing behavior, and mechanical modulus of asphalt. The simulations showed that paraffin significantly reduces the low-temperature stability, self-healing rate, and mechanical performance. Hu Q. et al. [68] used MD simulations to study the diffusion and thermodynamic behavior of warm mix recycled asphalt, showing that rejuvenators enhance its low-temperature performance by modifying its molecular structure and diffusion, whereas organic waxes require co-use with rejuvenators for optimal effectiveness.

### 3.3. Self-Healing Behavior of Asphalt Materials

The diffusion behavior of asphalt materials primarily includes processes such as asphalt self-healing and the mutual diffusion between rejuvenators and asphalt [7]. Molecular dynamics, as a microscopic method for investigating diffusion mechanisms, overcomes the difficulties of experimental research and the limitations of macroscopic theoretical analysis, thereby reproducing the diffusion process, especially the changes in the internal structure. The diffusion behavior of asphalt systems is generally characterized by the diffusion coefficient, expressed by the following formula:

(1) $$D=\frac{1}{6}\underset{t\to \infty }{\lim}\frac{d}{dt}\sum_{i=1}^{N}{\left[r_{i}(t)-r_{i}(0)\right]}^{2}$$

(2) $$MSD=\frac{1}{N}\sum_{i=1}^{N}{\langle r_{i}(t)-r_{i}(0)\rangle }^{2}$$

(3) $$D=\frac{\alpha }{6}$$

where *D* is the diffusion coefficient; *MSD* is the mean square displacement; *N* is the number of atoms; *r_i_*(*t*) and *r_i_*(0) represent the position vectors of particle *i* at time *t* and time 0, respectively. α represents the fitted slope of the MSD–time curve during the diffusive stage.

The diffusion coefficient is equal to one-sixth of the slope of the mean square displacement. By analyzing the motion trajectories of asphalt molecular structures under different ensemble conditions using molecular dynamics theory, the *MSD* can be obtained. Since molecular motion trajectories are related to the diffusion coefficient, the diffusion coefficient *D* can be calculated based on Equation (1). In Equation (3), the MSD curve becomes linear in the long-time diffusive regime, and its slope α can be used to simplify the calculation of the diffusion coefficient. This form is widely applied in practice, especially for analyzing the diffusion behavior of multi-component asphalt systems [78].

Fatigue cracking is one of the most common and critical failure modes in asphalt pavements. It not only directly affects the pavement service life and durability but may also induce the development of other types of distress. Studies have shown that introducing rest periods can effectively extend the fatigue life of asphalt mixtures, indicating that asphalt mixtures possess a certain degree of self-healing capability, which essentially originates from the intrinsic healing ability of asphalt materials [79]. To investigate the role of diffusion in asphalt self-healing, Luo et al. [80] established an asphalt–alumina interface model to study the diffusion behavior of asphalt components on the surface of alumina. The results showed that the diffusion rate of asphalt components is inversely related to their molecular mass, following the order: saturates > aromatics > resins > asphaltenes. In recent years, researchers worldwide [81,82,83,84,85,86,87] have conducted extensive studies on the self-healing behavior of asphalt binders and generally agree that molecular diffusion is the core mechanism underlying self-healing. This understanding provides a theoretical foundation for the durability-oriented design of asphalt pavements.

**Figure 24:**
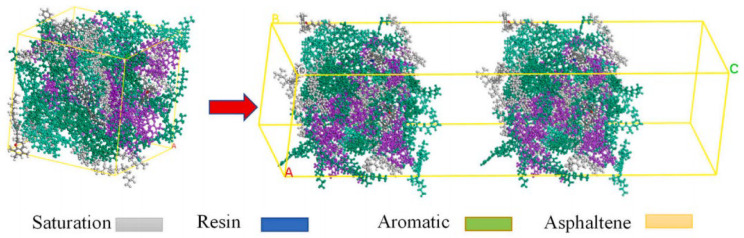
Self-healing model of asphalt [89].

With the continuous development of the molecular dynamics in the theoretical study of asphalt diffusion mechanisms, research on the self-healing behavior of asphalt has gradually deepened. Studies have shown that factors such as the crack width, temperature, type of base asphalt, and modifier content can significantly influence the self-healing performance of asphalt. Since the healing process primarily occurs at the crack interface, the healing mechanism can be investigated by simulating the diffusion behavior of asphalt molecules across the model layers [88]. A typical self-healing molecular model is constructed with a three-layer structure, in which asphalt molecular models are placed on both sides and a vacuum layer in the middle to simulate a microcrack. The self-healing capacity is assessed by observing and analyzing how asphalt molecules diffuse in opposite directions across the vacuum interface [78]. The construction process of the self-healing model is illustrated in Figure 24.

Traditional macro- and micro-scale experiments have not yet fully elucidated the thermal self-healing mechanisms of virgin, aged, and rejuvenated asphalt at the molecular level under different temperature conditions. Yu M. et al. [89] employed molecular dynamics along with macro- and micro-scale experiments to investigate the multiscale mechanisms of nanoscale crack healing in three types of asphalt. The results showed that during the long-range healing stage (0–40 ps), within the range of 60–80 Å, the relative concentration increased significantly from 0 mol/L to 0.6 mol/L, promoting rapid molecular reorganization around the crack and the formation of a self-healing network. Van der Waals forces were identified as the dominant driving force for the healing process. Wang S. et al. [90] examined the influence of the degradation level and content of crumb tire rubber (CTR) on the self-healing behavior of rubberized asphalt by controlling the degradation via screw extrusion. Highly degraded rubber (DCTR) exhibited the best flow healing capability due to its fine dispersion in asphalt and its highest loss factor at low temperatures. Yuan Y. et al. [91] used molecular dynamics modeling to explore the self-healing mechanism of terminal-blended (TB) composite modified asphalt and found that TB-SBS exhibited superior healing capability compared to other asphalt types, with significantly higher diffusion coefficients and collision frequencies. Bhasin A. et al. [78] utilized two types of binders with different molecular structures, demonstrating the practicality of molecular simulation in better understanding the relationships among molecular characteristics, healing mechanisms, and experimental parameters, which may be used to quantify these properties.

### 3.4. Aging Resistance of Asphalt

**Figure 25:**
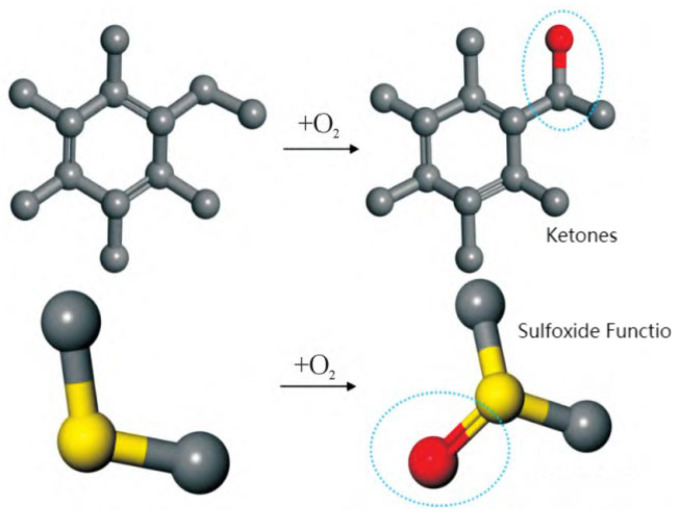
Formation of functional groups in aged asphalt [60].

The mechanisms of asphalt aging primarily involve oxidation, polymerization, and condensation processes, all of which have significant impacts on its performance. Oxidation is the primary cause of asphalt aging. During the oxidation process, oxygen replaces the hydrogen atoms attached to benzylic carbons, leading to the formation of ketones at these sites. This ultimately results in an increased concentration of ketones and sulfoxides, a reduction in the content of aromatic compounds and saturates, and an increase in asphaltenes. The complex aging process disrupts the originally stable colloidal structure of asphalt [92]. By introducing ketone and sulfoxide functional groups into base asphalt, an aged asphalt molecular model can be constructed to investigate the aging mechanism, as shown in Figure 25 [60]. In addition, oxidation reactions may also trigger polymerization and condensation reactions, leading to the formation of higher-molecular-weight products [93]. Polymerization is the process through which small molecules chemically react to form macromolecules. In the context of asphalt aging, such reactions result in an increase in molecular weight, leading to elevated viscosity and hardness. Consequently, the flexibility of asphalt decreases, making it more prone to cracking under low-temperature conditions. Furthermore, condensation is another important aging mechanism, involving chemical reactions between different components in asphalt that lead to the formation of new chemical structures. These reactions typically occur under high-temperature conditions, resulting in more complex molecular architectures and consequently increasing the hardness and viscosity of the asphalt [94]. Condensation reactions may also reduce the thermal stability of asphalt, making it more susceptible to softening or deformation under high-temperature conditions [95]. In summary, although multiple mechanisms contribute to asphalt aging, molecular dynamics studies have mainly concentrated on oxidative aging. Accordingly, this subsection focuses primarily on the oxidative aging behavior of asphalt.

**Figure 26:**
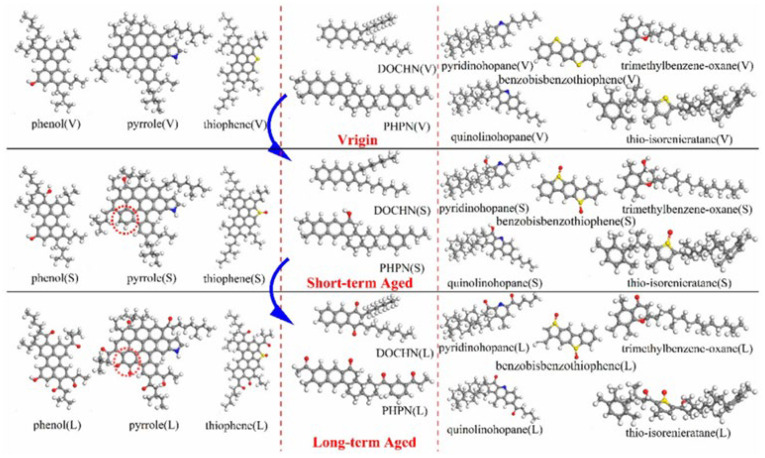
Short-term and long-term aging behavior of asphalt components [97].

Molecular dynamics can be used to investigate the aging mechanisms of asphalt binders. Results have shown that aged asphalt exhibits reduced free volume and increased density. The diffusion coefficients of asphaltenes and resins decrease at low temperatures, leading to reduced asphalt fluidity and, consequently, aging behavior [96]. Sun W. et al. [97] employed molecular dynamics simulations to analyze the aging behavior of a 12-component base asphalt model, categorizing the components into short-term and long-term aging stages, as illustrated in Figure 26.

**Figure 27:**
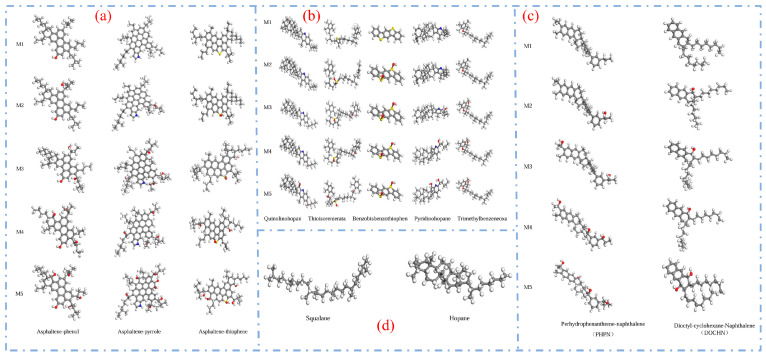
Molecular structures of five asphalt models: (**a**) asphaltene molecules (O: red; N: blue; S: yellow; C: gray; H: white); (**b**) resin molecules (O: red; N: blue; S: yellow; C: gray; H: white); (**c**) aromatic molecules (O: red; C: gray; H: white); (**d**) saturate molecules (C: gray; H: white) [98].

To further refine the understanding of short-term and long-term aging processes, Wang Qi [98] proposed a more detailed classification of asphalt aging, dividing the process into five stages (M1–M5). These stages correspond to the unaged, short-term aged, and long-term aged conditions described by Sun W. et al. [97]. Specifically, M1 represents the rapid aging initiation stage; M2 and M3 correspond to rapid aging molecular models; M4 represents the slow aging model; and M5 corresponds to the fully aged asphalt model. The aging models of the 12-component asphalt system at each of these five stages are illustrated in Figure 27.

**Figure 28:**
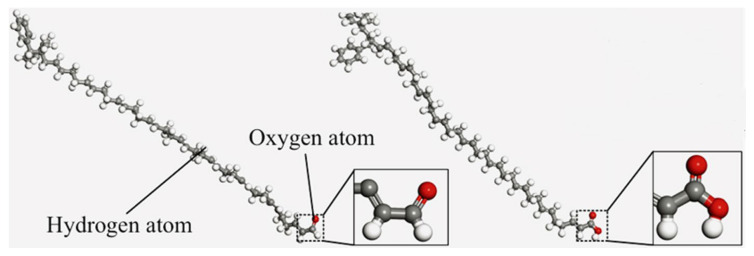
Aged SBS modifier molecular model [102].

During the long-term service of asphalt pavements, embrittlement caused by aging and the resulting reduction in mixture durability are the primary causes of pavement distress and cracking [99]. From a microscopic perspective, the aging of asphalt involves the following chemical changes: alterations in the molecular structure (chemical groups) of asphalt [100], changes in the molecular polarity and transformation of corresponding components [101], and variations in the average molecular weight and molecular weight distribution [102]. Based on the molecular models of base asphalt and SBS-modified asphalt, aging models can be regenerated by substituting atoms and chemical bonds at corresponding positions. The aging of SBS modifiers can also be categorized into short-term and long-term aging [103], as illustrated in Figure 28.

**Figure 29:**
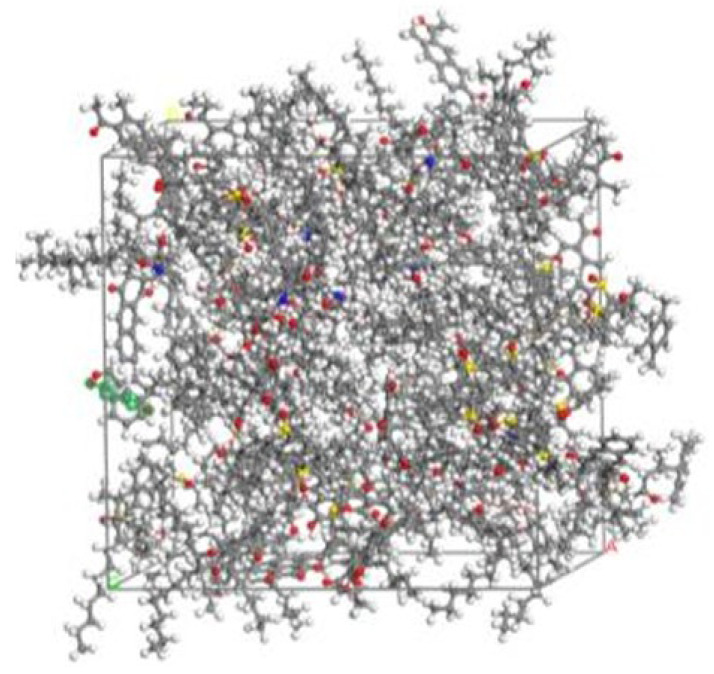
Aged SBS-modified asphalt molecular model [104].

**Figure 30:**
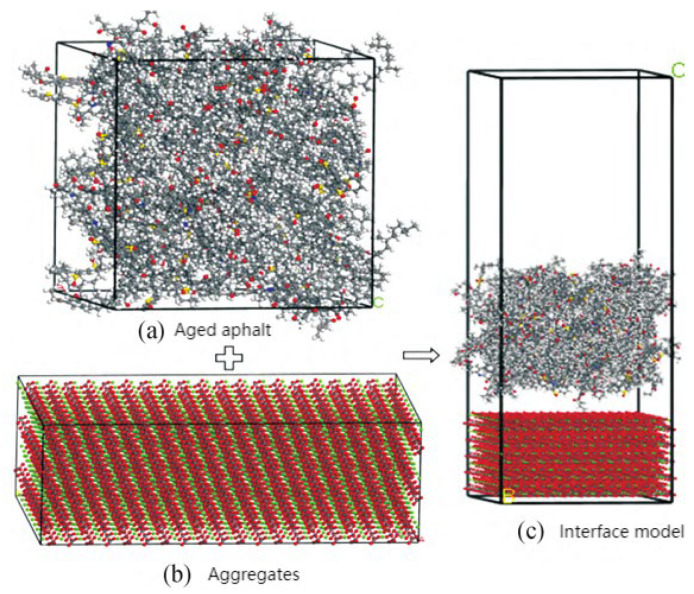
Adhesion and healing model of aged asphalt–aggregate interface [60].

Through calculation and optimization based on the molecular models of asphalt and SBS-modified asphalt, aged molecular models of SBS-modified asphalt have been obtained, as shown in Figure 29. Qiao Jiangang et al. [60] established an aged asphalt–aggregate model using the Build Layer function of MS software, as shown in Figure 30. Understanding the effects of asphalt aging is of great significance for the design of the service life and durability of asphalt materials. Xu G. et al. [100] used molecular dynamics simulations to study the effects of oxidative aging on asphalt’s self-healing potential and sensitivity to moisture damage. The results indicated that the presence of water molecules at the asphalt–aggregate interface leads to the degradation of adhesion work, which is more pronounced in aged asphalt. To investigate the mutual diffusion behavior between virgin and aged asphalt, Zhan Y. et al. [103] developed a composite molecular model of layered asphalt structures using MD. They found that at the same temperature, virgin asphalt exhibits the highest diffusion coefficient, followed by original aged asphalt and aged asphalt. As the temperature increases, there is a certain degree of overlap in the relative concentration on both sides of the aged asphalt.

**Figure 31:**
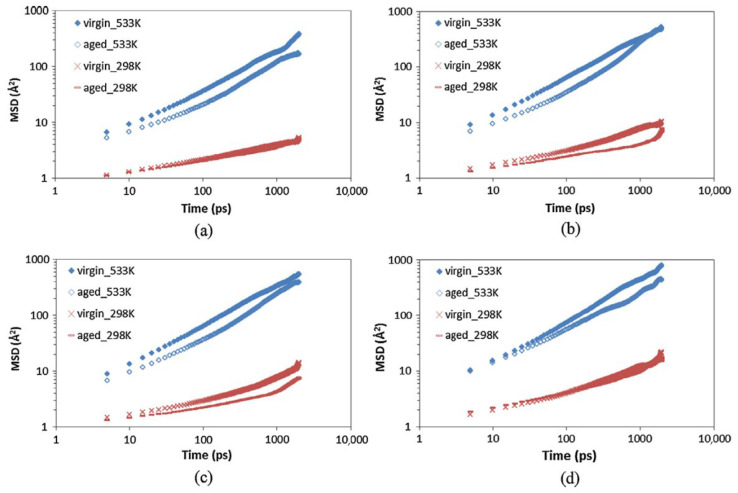
Comparison of the MSD of SARA fractions in virgin and aged asphalt for (**a**) asphaltene; (**b**) aromatic; (**c**) resin; and (**d**) saturate [100].

With the rapid development of road technologies and the widespread adoption of low-carbon transportation concepts, pavement recycling technology has gradually become a research focus. Among these technologies, rejuvenators, as key materials, play a crucial role in restoring the properties of aged asphalt. However, the effectiveness of rejuvenators largely depends on their diffusion depth within aged asphalt [105]. To further investigate the self-healing behavior of aged asphalt, Xu et al. [100] employed molecular dynamics simulations to study the effects of oxidative aging on asphalt. They constructed models representing both unaged and aged states at two different temperatures. Combined with mean square displacement (MSD) analysis, it was found that with increasing temperature and aging severity, aged asphalt exhibits a higher self-healing activation energy barrier compared to virgin asphalt (see Figure 31). Additionally, as the simulation time progresses, the diffusion coefficient of rejuvenators in the aged asphalt diffusion system gradually decreases; however, increasing temperature mitigates this decline. Further validation through rejuvenator-aged asphalt diffusion viscosity tests confirmed these findings, showing that temperature elevation significantly reduces the viscosity of aged asphalt, though the rate of viscosity reduction slows down as the temperature continues to rise. This indicates that the diffusion rate tends to decline at later stages, further supporting the time-dependent and temperature-sensitive nature of the rejuvenator diffusion process [106].

### 3.5. Adhesion Performance of the Asphalt–Aggregate Interface Model

The performance of asphalt pavements gradually deteriorates during long-term service, leading to various types of distress. When pavement damage reaches a certain level, maintenance of the existing pavement becomes necessary, with aggregate filling being one of the most critical repair methods [107]. Therefore, the composition of fine aggregate (fine and coarse fractions) and coarse aggregate (coarse fractions) as well as fillers must be carefully considered during the mix design of asphalt mixtures [108]. For the same type of asphalt, the lithology of the aggregate significantly influences the adhesion at the asphalt–aggregate interface and numerous related properties [109].

**Figure 32:**
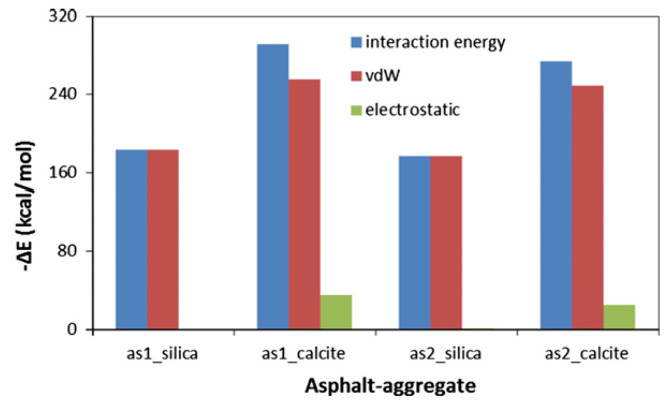
Interaction energy and its component predicted via MD simulation [115].

**Figure 33:**
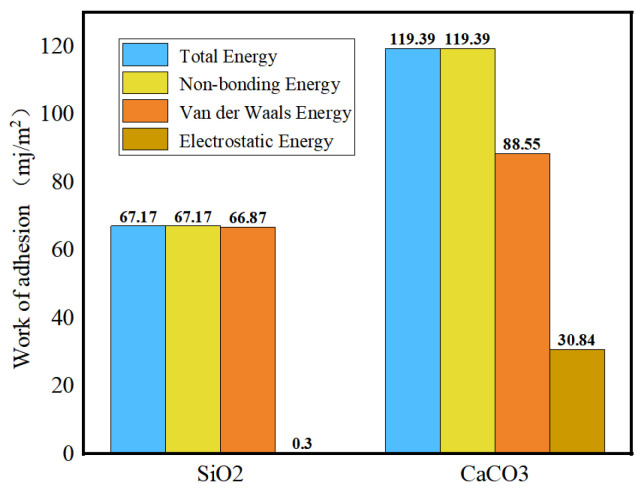
Work of adhesion between asphalt and aggregates [117].

**Figure 34:**
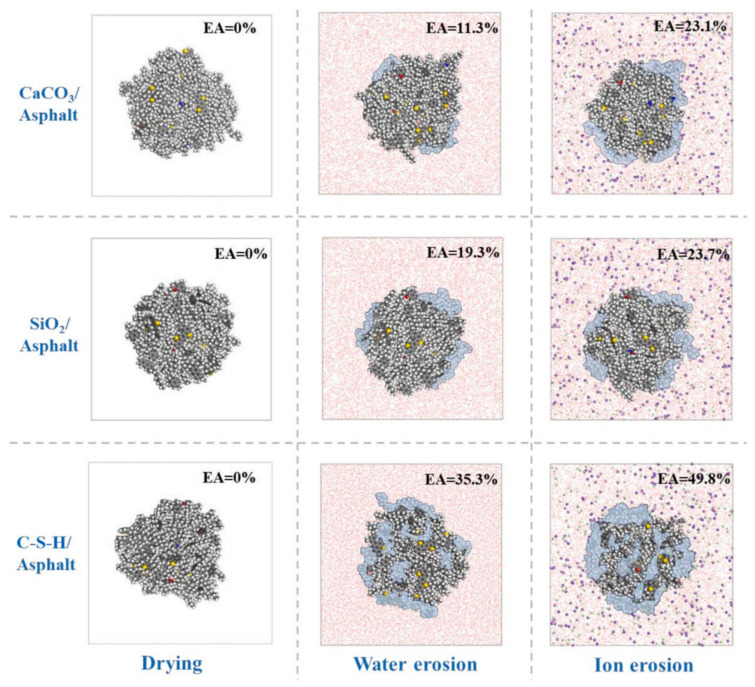
Percentage of water intrusion at the interface of asphalt and different aggregates [119].

The current research on the adhesion behavior at the asphalt–aggregate interface mainly focuses on two aspects: macroscopic experiments and microscopic simulations. In macroscopic experimental studies, the commonly used methods include surface energy tests [110,111], shear adhesion tests [112], and pull-off tests [113,114]. The adhesion between asphalt and aggregate is typically evaluated by reference to the interfacial energy. Xu G. et al. [115] investigated two types of asphalt and two types of aggregate. Their results showed that the interaction energy between asphalt and aggregate is primarily contributed by non-bonded interactions. Specifically, the adhesion between calcium oxide and asphalt is stronger than that between silica and asphalt, and alkaline aggregates exhibit better adhesion with asphalt than acidic aggregates (see Figure 32). Furthermore, the addition of modifiers significantly influences the adhesion strength at the asphalt–aggregate interface. For example, silane coupling agents (SCAs) improve adhesion under both dry and wet conditions by forming a thicker transition zone on the aggregate surface, thereby enhancing the compatibility between asphalt and aggregate, optimizing the force transfer mechanisms, and strengthening adhesion performance [116]. Lin Mei et al. [117] calculated the work of adhesion between base asphalt and two oxides (SiO_2_ and CaCO_3_). Their results (see Figure 33) indicated that the trends of adhesion work variation for both oxides are consistent and mainly dominated by non-bonded interactions, with bonded interactions contributing negligibly. Moreover, the adhesion work in the asphalt–SiO_2_ system is almost entirely provided by van der Waals forces, with a minimal contribution from electrostatic forces. In contrast, the adhesion work in the asphalt–CaCO_3_ system is significantly influenced by both van der Waals and electrostatic forces. Liu N. et al. [118] studied the adhesion characteristics of zeolite foam asphalt warm-mix mixtures (ZFA-WMMs), finding that adhesion failure is more likely with a high zeolite content or when using acidic aggregates such as quartz. Additionally, calcite exhibits stronger chemical adsorption capacity and better adhesion performance than quartz. Zhou J. et al. [119] further investigated the effects of moisture and chloride salt erosion on the bonding strength of recycled concrete aggregate (RCA)/asphalt interfaces, and simulated water damage on different aggregates using molecular dynamics (see Figure 34). Their results demonstrated that asphalt pavements containing RCA are susceptible to moisture and chloride salt erosion, necessitating effective mitigation measures.

## 4. Technical Challenges and Future Recommendations

Despite the substantial progress in applying molecular dynamics (MD) simulations to asphalt research, several key challenges remain, presenting critical opportunities for future investigation. The following directions are particularly worthy of attention:

(1) Model Realism and Experimental Integration

The current asphalt molecular models oversimplify the chemical complexity and dynamic variability under service conditions. Future work should focus on constructing more representative molecular models by incorporating diverse asphalt sources, compositional evolution during aging, and precise calibration using spectroscopic data (e.g., FTIR, NMR). Combining MD with experimental insights will significantly improve the model accuracy and simulation relevance.

(2) Multiscale and Efficient Simulation Strategies

Bridging the gap between molecular insights and macroscopic performance remains a core challenge. Future research should adopt multiscale approaches—such as coarse-grained MD, finite element methods, and machine learning-assisted frameworks—to enable cross-scale predictions. The development of parallel computing algorithms and AI-based acceleration tools will also be essential to reduce the computational costs and enhance the simulation efficiency.

(3) Chemical Reactivity, Modifier Behavior, and Interface Mechanics

Beyond physical properties, simulating chemical reactions such as aging, oxidation, and self-healing requires reactive force fields (e.g., ReaxFF) or hybrid QM/MM methods. Moreover, future studies should explore the compatibility, dispersion, and stability of polymer and nanomaterial modifiers under realistic service conditions. At the interface level, integrating MD with AFM, XPS, and SEM data can yield more accurate asphalt–aggregate adhesion models, especially in the presence of moisture and environmental degradation.

## 5. Conclusions

This paper reviews the molecular models of asphalt, modifiers, and asphalt–aggregate systems, covering developments from early average molecular models to the widely used four-component models, various common modifier models, and asphalt–aggregate molecular models. It provides an in-depth analysis of asphalt material properties from multiple perspectives, including mechanical performance, diffusion behavior, self-healing capability, and anti-aging performance, and summarizes the key research findings. A systematic analysis of the major achievements and technical challenges in the current studies is presented. The main conclusions are as follows:

(1) Asphalt is a complex multi-component material whose molecular structure and chemical composition vary significantly depending on the asphalt grade, the type of modifier, and the modification method. This variability poses a major challenge for the accurate construction of molecular models. The current modeling approaches primarily include average molecular models and molecular assembly methods, yet both still fall short of fully capturing the true microscopic composition of asphalt.
(2) Future research should focus on developing more refined, multiscale molecular models of asphalt and its modifiers to enhance the understanding of the roles of different chemical components at microscopic scales. Additionally, integrating experimental data for model optimization is essential to better reflect the actual composition and properties of asphalt materials.
(3) MD simulations offer advantages in studying the physical properties of asphalt—such as diffusion, self-healing, aging resistance, and interfacial adhesion—yet face challenges including high computational resource demands, limited simulation timescales, and difficulties in linking microscopic behavior to macroscopic mechanical properties. The current simulations predominantly focus on microscopic scales, and establishing effective correlations between molecular-level phenomena and macroscopic performance remains a key challenge.
(4) Future directions include advancing multiscale modeling techniques, such as combining coarse-grained MD (CGMD) with finite element methods (FEMs), to improve the simulation efficiency and achieve predictive capabilities spanning molecular to macroscopic levels. In asphalt–aggregate interface research, incorporating environmental factors (e.g., moisture, temperature, chemical corrosion) is necessary to enhance the model realism and applicability. Furthermore, coupling experimental data with high-resolution characterization techniques—such as X-ray photoelectron spectroscopy (XPS), atomic force microscopy (AFM), and scanning electron microscopy (SEM)—will improve the model reliability and engineering relevance.

## Figures and Tables

**Table 1:** Comparison of molecular assembly methods.

Comparison Items	Three-Component Model	Four-Component 12-Molecule Model
Model Composition	Maltenes, Resins, Asphaltenes	Aromatics, Saturates, Resins, Asphaltenes
Typicality	Highly generalizable and structurally concise	More accurately reflects the complex composition of asphalt
Advantages	The model is simple, computationally efficient, and suitable for preliminary simulation analysis.	The chemical composition is more comprehensive, enabling more accurate prediction of properties such as density and viscosity.
Disadvantages	Ignoring resins and aromatic fractions makes it difficult to reflect the structural diversity.	The model is relatively complex, with high construction difficulty and significant computational resource requirements.
Applicability	Preliminary study on the microstructure and behavior of asphalt.	In-depth study of structure–property relationships, self-healing, aging, and related aspects.
Quantity of representative molecules	Usually three types (e.g., C22, 1,7-dimethylnaphthalene, and asphaltenes).	Commonly consists of 12 types (e.g., quinoline, thiopyrene, etc.), with some models developed with up to 14 or 20 types.
Density matching accuracy	Moderate, with some deviations from the experimental values.	The density error can be controlled within 0.06 g/cm^3^, showing high agreement with the measured values.
Future scalability	Can be coupled with some modifiers, but with limited compatibility.	More suitable for incorporating complex systems such as SBS, aging molecules, and nanoparticles.

## Data Availability

Not applicable.
